# Accelerated Multiphosphorylated Peptide Synthesis

**DOI:** 10.1021/acs.oprd.2c00164

**Published:** 2022-07-12

**Authors:** Dana Grunhaus, Estefanía Rossich Molina, Roni Cohen, Tamar Stein, Assaf Friedler, Mattan Hurevich

**Affiliations:** The Institute of Chemistry, The Hebrew University of Jerusalem, Edmond J. Safra Campus, Givat Ram, Jerusalem, 91904, Israel

**Keywords:** peptide, phosphorylation, solid phase synthesis, acceleration, kinetics

## Abstract

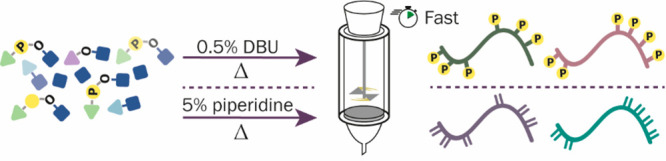

Preparing phosphorylated peptides with multiple adjacent phosphorylations is synthetically difficult, leads to β-elimination, results in low yields, and is extremely slow. We combined synthetic chemical methodologies with computational studies and engineering approaches to develop a strategy that takes advantage of fast stirring, high temperature, and a very low concentration of 1,8-diazabicyclo[5.4.0]undec-7-ene (DBU) to produce multiphosphorylated peptides at an extremely rapid time and high purity.

Phosphorylation plays an important role in regulating protein function in health and disease, and it is the most common post-translational modification. Most phosphoproteins are phosphorylated at multiple sites, creating clustered regions of phosphorylations.^1−4^ Since enzymatic phosphorylation of proteins leads to heterogeneous mixtures,^5^ the biological effect of phosphorylation patterns is usually studied using synthetic, homogeneous multiphosphorylated peptides (MPPs).^6−12^

Fmoc-based solid-phase peptide synthesis (SPPS) of MPPs with up to three phosphorylation sites is relatively straightforward in most cases.^13,14^ However, the synthesis of MPPs containing more than four phosphorylation sites (p-sites), especially clustered ones, is extremely difficult.^15^ First, the Fmoc-Ser/Thr(HPO_3_Bzl)-OH precursors used for the synthesis are sterically hindered and cause electrostatic repulsion, which decreases coupling yields. Second, protected phosphorylated Ser or Thr are prone to β-elimination under the alkaline conditions used for Fmoc deprotection (Figure 1).^16,17^ Manual and automated methods that enable the synthesis of MPPs rely on frequent adjustment of reaction conditions during the process and especially require systematic variation in the reaction temperature.^13−15^ This allowed the synthesis of MPPs with up to nine p-sites with minimal risk of β-elimination byproducts, but the process is very tedious and time-consuming.^18^ An average synthesis time of an MPP via one of the above methods ranges from several hours to days depending on the sequence and the phosphorylation pattern and is much slower than the synthesis of nonphosphorylated peptides.^19,20^ Accelerated SPPS processes are in high demand as peptide libraries are an essential tool for biological studies. Many strategies have been developed to produce peptides in minutes. These strategies could not be applied for MPP synthesis, because the high temperature, used for accelerating coupling reactions, promotes β-elimination of protected phosphorylated Ser/Thr during Fmoc deprotection (Figure 1).^21^

**Figure 1:**
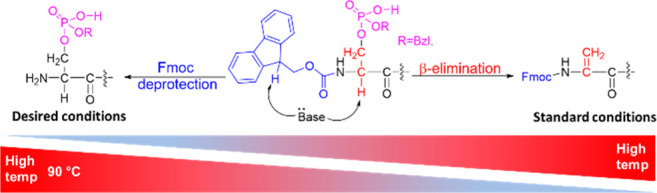
Fmoc deprotection (blue) and β-elimination (red) of protected phosphorylated Ser/Thr are competing reactions that take place under basic conditions. The common deprotection protocol, using 20% piperidine solution, results in complete deprotection at RT while promoting β-elimination at high temperature (right). New conditions are required for minimizing β-elimination during Fmoc deprotection at high temperatures (left).

The ability to shorten solid-phase synthesis reaction times is driven by the diffusion rate of the reagents and can be achieved by a combination of high temperature, efficient mixing, constant conditions, and a continuous process.^19,22,23^ We have recently introduced a setup that combines overhead stirring and constant high temperature for allowing accelerated SPPS at low reagent concentration (high-temperature fast-stirring peptide synthesis, HTFSPS).^23−25^ HTFSPS allows us to maximize diffusion dependent processes and enhance the efficiency of solid phase transformation in a short reaction time. HTFSPS was used for synthesizing short- to medium-size peptides of various levels of complexity in record time. Since HTFSPS proved its efficiency for various unmodified peptides, we assumed that it can be used also for the challenging synthesis of post translationally modified peptides like MPPs.

Here we took advantage of the high temperature and fast stirring setup to develop a method for accelerated multiphosphorylated peptide synthesis (AMPS) while minimizing β-elimination (Figure 1). Since MPPs suffer from several synthetic drawbacks, we tailored the chemistry to take advantage of the simplicity of HTFSPS while enabling the acceleration of the process. We looked for specific conditions that would enable removing Fmoc at a very short time and at high temperature without promoting β-elimination. We used those conditions to synthesize a library of MPPs derived from different phosphoproteins.

The type of base, its concentration, the reaction time, and the temperature can modulate the deprotection/elimination ratio. Hence, the study started with the search for a specific base and conditions that will allow for rapid Fmoc deprotection at high temperatures without promoting β-elimination (Figure 1).

**Figure 2:**
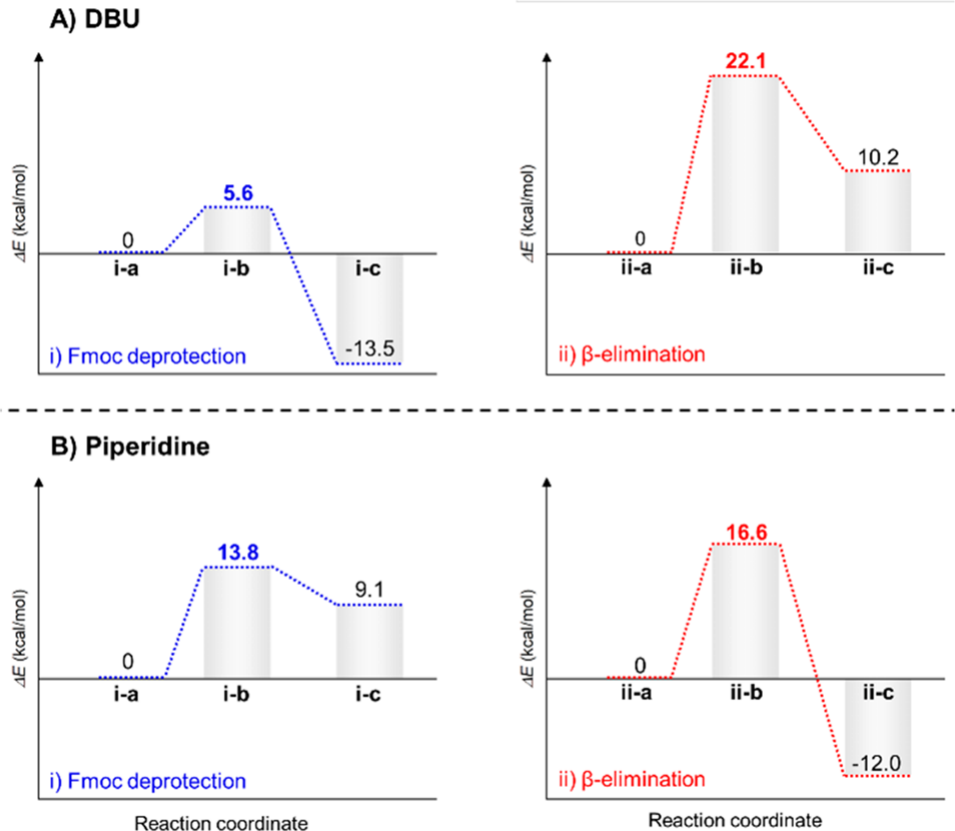
(A) Potential energy surface (PES) representing Fmoc deprotection (i) and β-elimination (ii) processes with DBU. (B) PES representing Fmoc deprotection (i) and β-elimination (ii) processes using piperidine. The chemical structure associated with the initial, final, and intermediate compounds, annotated i-a to i-c and ii-a to ii-c, are described in the SI. *ΔE* values are calculated with respect to the initial structures (i-a/ii-a).

The suppression of β-elimination using DBU, piperazine, and morpholine bases was previously demonstrated at 40 °C.^21^ 20% piperidine led to significant β-elimination at 40 °C, proving that this base cannot be used at the standard concentration under high temperatures. Since we aimed to perform the accelerated MPP synthesis at 90 °C using fast overhead stirring, we tested low concentrations of these four bases: 10% morpholine, 1% piperazine, 0.5% and 5% piperidine, and 0.5% DBU. These bases differ in their p*K*_b_ values and were all shown before to remove Fmoc at lower temperatures.^26−29^

We tested the ability to selectively remove Fmoc from the model phosphopeptide, Fmoc-pS(OBzl)LGLGLG (**Fmoc-a**), at 90 °C by treating the peptide with different base solutions for short, 5 min, and longer, 2 h, incubation periods. **Fmoc-a** contained Fmoc to assess the efficiency of its deprotection using different bases. It had a protected and phosphorylated pSer at its N terminal which is reported to be very sensitive toward β-elimination. In addition, a Leu-Gly linker was added to facilitate its purification. The efficiency and selectivity of the reactions were determined by HPLC (Table 1).

**Table 1:**
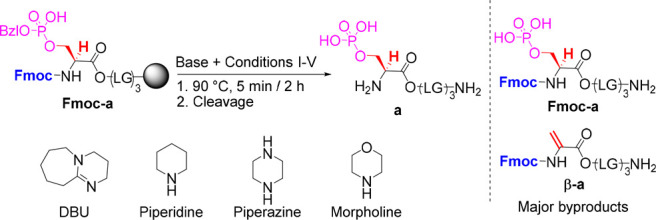
Screening for Optimal Fmoc Deprotection Conditions at 90 °C

		crude purity^c^ of **a** (%)	
conditions		5 min	2 h
I	0.5% DBU	96	97
II	0.5% Piperidine	65^b^	77^a^
III	5% Piperidine	28^b^	83^a^
IV	1% Piperazine	84^b^	72
V	10% Morpholine	67^a^,^b^	89^a^

a Incomplete deprotection.

b Significant β-elimination.

c Determined by HPLC.

Our results show that using 0.5% and 5% piperidine solutions resulted in incomplete deprotection after 5 min, and substantial formation of **β-a** and its piperidine adduct after 2 h. 10% Morpholine, the weakest base we tested, showed mainly incomplete Fmoc removal after 5 min, while leading to an elimination product after 2 h. Incubation with 1% piperazine resulted in low crude purity after 5 min and significant **β-a** byproduct after 2 h. 0.5% DBU solution provided almost complete Fmoc deprotection after 5 min with very high crude purity of **a**. No **β-a** was observed under these conditions even after 2 h at 90 °C. 0.5% DBU removed Fmoc, while suppressing β-elimination, making these specific conditions ideal for our setup. The high temperature and the overhead stirring both significantly improve the diffusion rate of the reaction,^22^ enabling the use of such a low DBU concentration for only several seconds with high efficiency.

p*K*_b_ values, steric hindrance, and other factors related to a specific base affect the efficiency of Fmoc deprotection and the selectivity compared to the competing elimination process. We used theoretical models to rationalize the observed differences. To understand the mechanisms of Fmoc deprotection and β-elimination at a molecular level, the reactions with either DBU (Figure 2) or with piperidine were modeled using density functional theory (DFT).^30^

The minima and transition states on the potential energy surfaces (PESs) were identified, and the energy barriers for the studied reactions were determined (the detailed methodology is given in the Experimental Section). As a model system, we used a Fmoc-Ser/(HPO_3_Bzl)-OH as it has the relevant molecular features of a phosphorylated amino acid in the peptide and its size simplifies calculations. The PESs of Fmoc deprotection and β-elimination via DBU were calculated independently. The Fmoc deprotection mechanisms proceed in two stages (see SI). The most important transition involves dibenzofulvene abstraction (i-a to i-c) that consists of the high-energy intermediate i-b. The proton transfer from the cyclopentyl moiety of the Fmoc group to the DBU leads to the formation of the i-b transition state with a barrier of 5.6 kcal/mol.

To model the β-elimination reaction, a proton is transferred from the Cα of the [Ser/(PO_3_Bzl)-OH]^−^ to the DBU ii-a via the 22.1 kcal/mol transition state ii-b, which leads to the dephosphorylation giving the dihydroalanine product ii-c. These results show that β-elimination using DBU requires 16 kcal/mol more than Fmoc deprotection, which makes Fmoc deprotection kinetically favorable.

PESs for the same reactions performed using piperidine as a base showed that the energy barriers for Fmoc deprotection (Figure 2bi) and β-elimination (2bii) are comparable (described in the Experimental Section in the SI). Calculating the energies of the transition states for Fmoc deprotection and β-elimination reactions using either DBU or piperidine as bases allowed us to rationalize the difference in selectivity observed in the experimental model (Figure 2).

Taken together, the calculations showed that with piperidine used as a base, the energy barriers are similar (∼3 kcal/mol difference), supporting the observation that at high temperature, elimination competes with deprotection. In the case of the DBU, the barrier for the β-elimination is substantially higher than the one leading to deprotection, which explains the experimental observation that Fmoc deprotection is energetically favorable over the β-elimination. The methodologic study and theoretical calculations suggest that 0.5% DBU can remove Fmoc without causing major β-elimination at 90 °C.

An MPP derived from B2 bradykinin receptor 366–375, **B2R-5p**, was selected as a biological model, as it has five clustered p-sites at its C-terminus that contain both pSer and pThr residues (Table 2).^31^ To compare to the state-of-the-art, we first attempted the synthesis of **B2R-5p** by a phosphopeptide-specific MW-assisted strategy. Couplings under MW were performed using 5 equiv of protected amino acid at 75 °C for 10 min using 1-[bis(dimethylamino)methylene]-1*H*-1,2,3-triazolo[4,5-*b*]pyridinium 3-oxide hexafluorophosphate (HATU)/*N*,*N*-diisopropylethyl amine (DIEA). It was followed with five DMF washing cycles to cool down the resin before the deprotection step at RT. The deprotection was then performed using 20% piperidine without MW for 10 min. The entire MW-assisted process took over six hours and resulted in no formation of **B2R-5p** (Figure 3A and SI).

**Figure 3:**
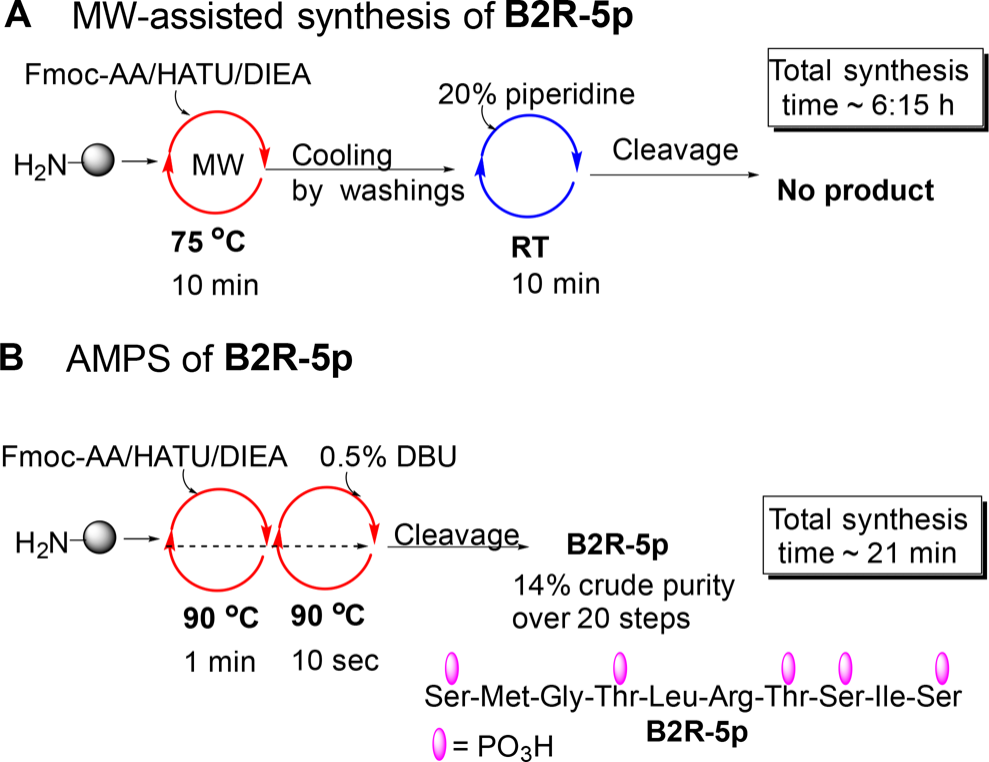
**B2R-5p** synthesis by either the MW-assisted (A) or AMPS (B) approaches. In both syntheses, three residues (marked with *) were coupled twice pSMGpT*LR*pT*pSIpS since the coupling involved an introduction of a bulky pThr or Arg to clustered regions_._.

**Table 2:** Library of Multiphosphorylated Peptides Synthesized in the Current Study

entry	protein region	sequence	crude purity [%]	yield [%]^a^
V2R-5p	V2R (362–371)	pSpSpSLAKDpTpSS	7.7	4.2
APC-4p	APC (1502–1511)	CpS*pS*SLpSALpSL	31.5	27.0
p53–5p	P53 (6–20)	pS*DPpS*VEPPLpSQEpT*FpS*	19.9	6.5
B2R-5p	B2 bradykinin receptor (366–375)	pSMGpT*LR*pT*pSIpS	13.7	12.8
Vim-4p	Vimentin (S22–S29)	pS*R*P*pS*pS*pSRSLLL^b^	27.8	24.0
Tau-6p	Tau (515–527)	pS*pSPGpSPG*pT*PGpS*R*pS*LLL^b^	12.7	3.6
pLam-4p	preLamin A/C (404–411)	pS*HpS*pSQ*pT*QGLLL^b^	37.2	33.6
FFA-5p	Free Fatty Acid receptor 4 (346–360)	L*pT*D*pT*pS*VKRNDLpSIIpS	14.2	6.9

a The yields were determined by the mass of an isolated pure peptide divided by the mass of a crude peptide. Crude purity was determined by HPLC from the ratio between the peak of the desired MPP and the sum of all other integration peaks.

b Peptides with large number of p-sites and/or polar residues were added to a non-native hydrophobic tri-Leu sequence to facilitate purification (blue).

We next synthesized **B2R-5p** using the AMPS method that included (i) constant heating at 90 °C, (ii) fast and constant overhead stirring at 1200 rpm, and (iii) short reaction and washing cycles.^23^ We used 0.5% DBU solution for 10 s to minimize β-elimination. Couplings were performed with 3 equiv AA for 1 min using HATU/DIEA. AMPS of **B2R-5p** took only 21 min and resulted in a crude purity of 13.7% and in the isolation of 2.5 mg pure peptide (Figure 3; for **B2R-5p** chromatograms and ESI-MS see SI). Considering the difficulty of the synthesis and the short synthetic process, the yields obtained are satisfactory. The comparative study proved that AMPS is superior to the MW-assisted method in the synthesis of MPPs.

One of the biggest advantages of rapid peptide synthesis is the ability to synthesize peptide libraries. To demonstrate the applicability of AMPS we synthesized a library of MPPs derived from phosphorylated regions of eight different proteins:

The library included Vasopressin V2 receptor 362–371 (**V2R-5p**),^32^ Adenomatous polyposis coli protein 1502–1511 (**APC-4p**),^33^ Tau 515–527 (**Tau-6p**),^34^ Free fatty acid receptor 4 346–360 (**FFAR4-5p**),^35^ Cellular tumor antigen p53 6–20 (**p53-5p**),^36^ Vimentin 22–29 (**Vim-4p**),^37^ Prelamin-A/C 404–411 (**PLam-4p**).^38^ All the selected MPPs contain at least four phosphorylation sites (Table 2).

Couplings in AMPS were performed for 1 min with the HATU/DIEA activating system. His and Cys were coupled twice for 30 s. Deprotection was performed using 0.5% DBU in DMF for 10 s. All peptides were cleaved from the resin using a standard TFA cocktail for 5 to 7 h depending on the number of p-sites, purified using HPLC, and lyophilized before the yield and purity of the isolated peptide were determined. In all cases, RP-HPLC purification provided a mg scale of the pure MPP. AMPS was applied successfully for synthesizing a library of eight MPPs with varied sequences and phosphorylation patterns.

The resulting crude purities of MPPs synthesized by AMPS, ranged between 7.7% and 37.2%. The overall yields ranged between 3.6% and 33.6%, resulting in a few milligrams of >95% pure MPPs. The clustering of phosphorylated amino acids, their overall number, and the specific pThr/pSer combination all affect the crude purity (Table 2). In addition, the peptide sequence and the presence of other bulky/hindered amino acids also contribute to the difficulty in synthesis which translates to lower purity. With these factors taken into account, the differences in purities between the peptides are easy to explain. The most important fact is that each MPP we tried to synthesize was isolated in high purity and sufficient quantities.

We previously showed that the targeted and fully automated approaches allow fine-tuning of every step of the MPP synthesis. The automated approach provides MPPs with five p-sites in crude purity of above 50%, and the targeted approach can provide even higher purities.^15,18^ Although crude purities obtained by those methods are higher than AMPS, a synthesis of 10 residues in the Glyconeer 2.1 takes ∼14 h, and via the targeted approach it takes days.^15,18^ This makes the synthesis of each MPP about 40 times longer than via AMPS. Both these methods suffer from poor mixing of the solid support and the reaction reagents, whether by shaking or gas bubbling which translates to a longer reaction duration in addition to the cooling–heating cycles that are applied. The constant stirring and high temperature used in AMPS make each step of the process much faster, thus enabling MPP synthesis in minutes.

The differences between the MPP synthesis strategies imply that they should be used for different purposes. The targeted and the automated approaches are excellent choices when a single MPP is to be synthesized in high purity and yield. AMPS is the preferred choice when a series of MPPs are to be synthesized in a short time to allow biological screening. This suggests that AMPS will have a unique place in the MPP synthetic strategies toolbox.^13,14^

While highly attractive and already applicable for nonphosphorylated peptides, the accelerated synthesis of MPPs to date is practically nonexistent. We present herein a new method for rapid MPP synthesis and its application for synthesizing a diverse MPP library. The experimentally driven and theoretically supported choice-of-base allowed us to maximize Fmoc removal while minimizing β-elimination, thus overcoming a major hurdle in MPP synthesis. The overhead stirring enabled us to decrease the duration of reactions and the reagent concentrations. Altogether, an accelerated synthesis approach was applied for the synthesis of highly valuable yet mostly inaccessible multiphosphorylated peptides. We showed here that by changing the conditions of the synthesis HTFSPS can be adjusted for synthesizing peptides with post-transitional modifications, thus opening the route for the accelerated synthesis of other types of modified peptides. By changing the design of the reactor we are certain that the advantages of HTFSPS can be adopted for accelerate scaled-up peptide synthesis.

## Experimental Section

### Essential Synthesis and Base Screening Protocol

A short model peptide Fmoc-pSer(OBzl)LGLGLG (**Fmoc-a**) was synthesized. 100 mg of resin was swelled for 30 min in ∼5 mL of DMF, using 2.9 equiv HATU and 8 equiv of DIEA as the activating system and 20% piperidine/DMF for Fmoc deprotection; final Fmoc deprotection was not performed. Coupling steps duration was 1 min, and deprotection steps were 30 s. The resin was washed thoroughly by 3 × 5 mL DMF, 3 × 5 mL DCM, and 3 × 5 mL MeOH and dried carefully. The peptide-bound resin was divided into 12 test tubes with 10 mg in each tube. Then 1 mL from one of the following base solutions was added for 2 test tubes from each condition: 0.5% and 5% of piperidine, 0.5% of DBU, 1% of piperazine, and 10% of morpholine. Immediately after adding the base, samples were incubated inside a 90 °C water bath. One set of samples was incubated for 5 min and the second set of samples incubated for 2 h. The resin of each sample was transferred to a small-fritted tube, washed with 3 × 5 mL DMF, 3 × 5 mL DCM, and 3 × 5 mL MeOH and dried carefully. Peptides were cleaved, and the crude material was dissolved in 200 μL TDW and 50 μL ACN and filtered, and 80 μL was injected into an analytical RP-HPLC. Samples were eluted using a 5–60% ACN gradient, and the signal was recorded at 220 nm. Peaks were collected and analyzed by electron spray ionization mass spectrometry (ESI-MS). Crude purity was calculated by peak integration; the area under the peak of the deprotected peptide was divided by the sum of all integration values in the relevant range.

### Procedure for AMPS

A reactor containing a sintered glass filter and a heating jacket was used. The heating jacket was connected to a circulating 90 °C water bath. The reactor has a narrow diameter to enable fast and efficient heat transfer. The reactor was equipped with an overhead 5-fin turbine PTFE impeller. Solvents and reagents were inserted directly by a feeding line and drained by vacuum filtration. 3 mL of coupling mixture containing 3 equiv of protected amino acid, 2.9 equiv of HATU, and 8 equiv of DIEA was added to the reactor without preactivation or preheating. Fmoc deprotection was done using 3 mL of a 0.5% DBU solution in DMF without preheating. Peptides were cleaved according to the procedure above.

### Peptide Cleavage from the Resin

A freshly prepared solution (6 mL) of trifluoroacetic acid (TFA)/triisopropylsilane (TIPS)/TDW (92:3:5) was added to 100 mg of dry peptidyl-resin. The mixture was shaken at room temperature between 5 and 7 h depending on the number of phosphorylated residues. Then, the resin was separated by filtration. The TFA was removed under nitrogen atmosphere, and the peptide was precipitated by gradual addition of ice-cold ether to the mixture. The solution was centrifuged, and the peptide was washed twice with ether. A minimum volume of TDW was used to dissolve the crude peptide, which was then lyophilized to dryness before HPLC purification and ESI-MS analysis.

### Computational Details

All density functional theory (DFT)^30^ calculations in this study were performed using Q-Chem software, version 5.4.^39^ We utilized the B3LYP exchange-correlation functional,^40^ combined with D3 Grimme correction for dispersion forces, which accounts for the noncovalent interactions, crucial for the systems under study.^41^ The calculations were done using the 6-31+G* basis set.^42^ Initial guesses for the transition states were obtained using the Freezing String Method (FSM).^43^ The guess-structures were further optimized to obtain the transition states of the system. Intrinsic Reaction Coordinate (IRC)^44^ was used to verify that the calculated transition states indeed connect the relevant reactants and products. In the cases where the IRC calculations did not converge, we manually modified the transition state structure slightly along with its imaginary normal mode and verified that, upon optimization, these structures led to the reactants and products of interest.

## Supporting Information Available

Additional experimental details, materials, and methods, including detailed DFT calculation and chemical structures of all compounds in studied reactions. HPLC chromatograms and ESI-MS of peptide library. (PDF)

## References

[ref1] Salazar C.; Höfer T. Multisite Protein Phosphorylation - From Molecular Mechanisms to Kinetic Models. FEBS J. 2009, 276 (12), 3177–3198. 10.1111/j.1742-4658.2009.07027.x. 19438722

[ref2] Valk E.; Venta R.; Örd M.; Faustova I.; Kõivomägi M.; Loog M. Multistep Phosphorylation Systems: Tunable Components of Biological Signaling Circuits. Mol. Biol. Cell 2014, 25, 3437–3716. 10.1091/mbc.e14-02-0774. 25368420 PMC4230602

[ref3] Moses A. M.; Hériché J. K.; Durbin R. Clustering of Phosphorylation Site Recognition Motifs Can Be Exploited to Predict the Targets of Cyclin-Dependent Kinase. Genome Biol. 2007, 8, R23 10.1186/gb-2007-8-2-r23. 17316440 PMC1852407

[ref4] Schweiger R.; Linial M. Cooperativity within Proximal Phosphorylation Sites Is Revealed from Large-Scale Proteomics Data. Biol. Direct 2010, 5 (6), 6 10.1186/1745-6150-5-6. 20100358 PMC2828979

[ref5] Slovakova M.; Bilkova Z. Contemporary Enzyme-Based Methods for Recombinant Proteins in Vitro Phosphorylation. Catalysts 2021, 11, 1007 10.3390/catal11081007.

[ref6] Ardito F.; Giuliani M.; Perrone D.; Troiano G.; Muzio L. lo. The Crucial Role of Protein Phosphorylation in Cell Signalingand Its Use as Targeted Therapy (Review). Int. J. Mol. Med. 2017, 40 (2), 271–280. 10.3892/ijmm.2017.3036. 28656226 PMC5500920

[ref7] Lim W. A.; Pawson T. Phosphotyrosine Signaling: Evolving a New Cellular Communication System. Cell 2010, 142, 661–667. 10.1016/j.cell.2010.08.023. 20813250 PMC2950826

[ref8] Mayer D.; Damberger F. F.; Samarasimhareddy M.; Feldmueller M.; Vuckovic Z.; Flock T.; Bauer B.; Mutt E.; Zosel F.; Allain F. H. T.; Standfuss J.; Schertler G. F. X.; Deupi X.; Sommer M. E.; Hurevich M.; Friedler A.; Veprintsev D. B. Distinct G Protein-Coupled Receptor Phosphorylation Motifs Modulate Arrestin Affinity and Activation and Global Conformation. Nat. Commun. 2019, 10, 1261 10.1038/s41467-019-09204-y. 30890705 PMC6424980

[ref9] Despres C.; Byrne C.; Qi H.; Cantrelle F. X.; Huvent I.; Chambraud B.; Baulieu E. E.; Jacquot Y.; Landrieu I.; Lippens G.; Smet-Nocca C. Identification of the Tau Phosphorylation Pattern That Drives Its Aggregation. Proc. Natl. Acad. Sci. U. S. A. 2017, 114, 9080–9085. 10.1073/pnas.1708448114. 28784767 PMC5576827

[ref10] Kaya A. I.; Perry N. A.; Gurevich V. v; Iverson T. M. Phosphorylation Barcode-Dependent Signal Bias of the Dopamine D1 Receptor. Proc. Natl. Acad. Sci. U. S. A. 2020, 117, 14139–14149. 10.1073/pnas.1918736117. 32503917 PMC7321966

[ref11] Latorraca N. R.; Masureel M.; Hollingsworth S. A.; Heydenreich F. M.; Suomivuori C. M.; Brinton C.; Townshend R. J. L.; Bouvier M.; Kobilka B. K.; Dror R. O. How GPCR Phosphorylation Patterns Orchestrate Arrestin-Mediated Signaling. Cell 2020, 183, 1813–1825. 10.1016/j.cell.2020.11.014. 33296703 PMC7901245

[ref12] Li Y.; Heng J.; Sun D.; Zhang B.; Zhang X.; Zheng Y.; Shi W.-W.; Wang T.-Y.; Li J.-Y.; Sun X.; Liu X.; Zheng J.-S.; Kobilka B. K.; Liu L. Chemical Synthesis of a Full-Length G-Protein-Coupled Receptor Β2-Adrenergic Receptor with Defined Modification Patterns at the C-Terminus. J. Am. Chem. Soc. 2021, 143, 17566–17576. 10.1021/jacs.1c07369. 34663067

[ref13] Harris P. W. R.; Williams G. M.; Shepherd P.; Brimble M. A. The Synthesis of Phosphopeptides Using Microwave-Assisted Solid Phase Peptide Synthesis. Int. J. Pept Res. Ther 2008, 14 (4), 387–392. 10.1007/s10989-008-9149-9.

[ref14] Qvit N. Microwave-Assisted Synthesis of Cyclic Phosphopeptide on Solid Support. Chem. Biol. Drug Des 2015, 85, 300–305. 10.1111/cbdd.12388. 25042903

[ref15] Samarasimhareddy M.; Mayer D.; Metanis N.; Veprintsev D.; Hurevich M.; Friedler A. A Targeted Approach for the Synthesis of Multi-Phosphorylated Peptides: A Tool for Studying the Role of Phosphorylation Patterns in Proteins. Org. Biomol. Chem. 2019, 17 (42), 9284–9290. 10.1039/C9OB01874C. 31497840

[ref16] Attard T. J.; O’Brien-Simpson N.; Reynolds E. C. Synthesis of Phosphopeptides in the Fmoc Mode. Int. J. Pept Res. Ther 2007, 13, 447–468. 10.1007/s10989-007-9107-y.

[ref17] Yang Y. β-Elimination Side Reactions. In Side Reactions in Peptide Synthesis; Academic Press: Cambridge, 2016; pp 33–42. 10.1016/b978-0-12-801009-9.00002-1.

[ref18] Grunhaus D.; Friedler A.; Hurevich M. Automated Synthesis of Heavily Phosphorylated Peptides. Eur. J. Org. Chem. 2021, 2021, 3737–3742. 10.1002/ejoc.202100691.

[ref19] Mijalis A. J.; Thomas D. A.; Simon M. D.; Adamo A.; Beaumont R.; Jensen K. F.; Pentelute B. L. A Fully Automated Flow-Based Approach for Accelerated Peptide Synthesis. Nat. Chem. Biol. 2017, 13 (5), 464–466. 10.1038/nchembio.2318. 28244989

[ref20] Simon M. D.; Heider P. L.; Adamo A.; Vinogradov A. A.; Mong S. K.; Li X.; Berger T.; Policarpo R. L.; Zhang C.; Zou Y.; Liao X.; Spokoyny A. M.; Jensen K. F.; Pentelute B. L. Rapid Flow-Based Peptide Synthesis. ChemBioChem. 2014, 15 (5), 713–720. 10.1002/cbic.201300796. 24616230 PMC4045704

[ref21] Attard T. J.; O’Brien-Simpson N. M.; Reynolds E. C. Identification and Suppression of β-Elimination Byproducts Arising from the Use of Fmoc-Ser(PO3Bzl,H)-OH in Peptide Synthesis. Int. J. Pept Res. Ther 2009, 15, 69–79. 10.1007/s10989-008-9165-9.

[ref22] Rosenau P. Reaction and Concentration Dependent Diffusion Model. Phys. Rev. Lett. 2002, 88, 194501 10.1103/PhysRevLett.88.194501. 12005636

[ref23] Naoum J. N.; Alshanski I.; Mayer G.; Strauss P.; Hurevich M. Stirring Peptide Synthesis to a New Level of Efficiency. Org. Process Res. Dev 2022, 26, 129–136. 10.1021/acs.oprd.1c00304.

[ref24] Alshanski I.; Bentolila M.; Gitlin-Domagalska A.; Zamir D.; Zorsky S.; Joubran S.; Hurevich M.; Gilon C. Enhancing the Efficiency of the Solid Phase Peptide Synthesis (SPPS) Process by High Shear Mixing. Org. Process Res. Dev 2018, 22, 1318–1322. 10.1021/acs.oprd.8b00225.

[ref25] Pedersen S. L.; Tofteng A. P.; Malik L.; Jensen K. J. Microwave Heating in Solid-Phase Peptide Synthesis. Chem. Soc. Rev. 2012, 41 (5), 1826–1844. 10.1039/C1CS15214A. 22012213

[ref26] Ralhan K.; KrishnaKumar V. G.; Gupta S. Piperazine and DBU: A Safer Alternative for Rapid and Efficient Fmoc Deprotection in Solid Phase Peptide Synthesis. RSC Adv. 2015, 5, 104417–104425. 10.1039/C5RA23441G.

[ref27] Lobo-Ruiz A.; Tulla-Puche J. General Fmoc-Based Solid-Phase Synthesis of Complex Depsipeptides Circumventing Problematic Fmoc Removal. Eur. J. Org. Chem. 2020, 2020, 183–192. 10.1002/ejoc.201901459.

[ref28] Luna O.; Gomez J.; Cárdenas C.; Albericio F.; Marshall S.; Guzmán F. Deprotection Reagents in Fmoc Solid Phase Peptide Synthesis: Moving Away from Piperidine?. Molecules 2016, 21 (11), 1542 10.3390/molecules21111542. PMC6274427 27854291

[ref29] Li W.; O’Brien-Simpson N. M.; Hossain M. A.; Wade J. D. The 9-Fluorenylmethoxycarbonyl (Fmoc) Group in Chemical Peptide Synthesis – Its Past, Present, and Future. Aust. J. Chem. 2020, 73 (4), 271–276. 10.1071/CH19427.

[ref30] Parr R. C.; Yang W. Density-Functional Theory of the Electronic Structure of Molecules. Annu. Rev. Phys. Chem. 1995, 46, 701–729. 10.1146/annurev.pc.46.100195.003413. 24341393

[ref31] Blaukat A.; Pizard A.; Breit A.; Wernstedt C.; Alhenc-Gelas F.; Müller-Esterl W.; Dikic I. Determination of Bradykinin B2 Receptor in Vivo Phosphorylation Sites and Their Role in Receptor Function. J. Biol. Chem. 2001, 276 (44), 40431–40440. 10.1074/jbc.M107024200. 11517230

[ref32] He Q.-T.; Huang S.-M.; Jia Y.-L.; Zhu Z.-L.; Lin J.-Y.; Yang F.; Tao X.-N.; Zhao R.-J.; Gao F.-Y.; Niu X.-G.; Xiao K.-H.; Wang J.; Jin C.; Sun J.-P.; Yu X. Structural Studies of Phosphorylation-Dependent Interactions between the V2R Receptor and Arrestin-2. Nat. Commun. 2021, 12, 1–16. 10.1038/s41467-021-22731-x. 33888704 PMC8062632

[ref33] Xing Y.; Clements W. K.; le Trong I.; Hinds T. R.; Stenkamp R.; Kimelman D.; Xu W. Crystal Structure of a β-Catenin/APC Complex Reveals a Critical Role for APC Phosphorylation in APC Function. Mol. Cell 2004, 15, 523–533. 10.1016/j.molcel.2004.08.001. 15327769

[ref34] Sato S.; Cerny R. L.; Buescher J. L.; Ikezu T. Tau-Tubulin Kinase 1 (TTBK1), a Neuron-Specific Tau Kinase Candidate, Is Involved in Tau Phosphorylation and Aggregation. J. Neurochem. 2006, 98, 1573–1584. 10.1111/j.1471-4159.2006.04059.x. 16923168

[ref35] Butcher A. J.; Hudson B. D.; Shimpukade B.; Alvarez-Curto E.; Prihandoko R.; Ulven T.; Milligan G.; Tobin A. B. Concomitant Action of Structural Elements and Receptor Phosphorylation Determines Arrestin-3 Interaction with the Free Fatty Acid Receptor FFA4. J. Biol. Chem. 2014, 289 (26), 18451–18465. 10.1074/jbc.M114.568816. 24817122 PMC4140278

[ref36] Jenkins L. M. M.; Durell S. R.; Mazur S. J.; Appella E. P53 N-Terminal Phosphorylation: A Defining Layer of Complex Regulation. Carcinogenesis 2012, 33 (8), 1441–1449. 10.1093/carcin/bgs145. 22505655 PMC3499055

[ref37] Izawa I.; Inagaki M. Regulatory Mechanisms and Functions of Intermediate Filaments: A Study Using Site- and Phosphorylation State-Specific Antibodies. Cancer Sci. 2006, 97, 167–174. 10.1111/j.1349-7006.2006.00161.x. 16542212 PMC11159468

[ref38] Torvaldson E.; Kochin V.; Eriksson J. E. Phosphorylation of Lamins Determine Their Structural Properties and Signaling Functions. Nucleus 2015, 6 (3), 166 10.1080/19491034.2015.1017167. 25793944 PMC4615644

[ref39] Epifanovsky E.; Gilbert A. T. B.; Feng X.; Lee J.; Mao Y.; Mardirossian N.; Pokhilko P.; White A. F.; Coons M. P.; Dempwolff A. L.; et al. I. Software for the Frontiers of Quantum Chemistry: An Overview of Developments in the Q-Chem 5 Package. J. Chem. Phys. 2021, 155 (8), 084801 10.1063/5.0055522. 34470363 PMC9984241

[ref40] Becke A. D. A New Mixing of Hartree–Fock and Local Density-Functional Theories. J. Chem. Phys. 1993, 98, 1372 10.1063/1.464304.

[ref41] Grimme S.; Antony J.; Ehrlich S.; Krieg H. A Consistent and Accurate Ab Initio Parametrization of Density Functional Dispersion Correction (DFT-D) for the 94 Elements H-Pu. J. Chem. Phys. 2010, 132, 154104 10.1063/1.3382344. 20423165

[ref42] Ditchfield R.; Hehre W. J.; Pople J. A. Self-Consistent Molecular-Orbital Methods. IX. An Extended Gaussian-Type Basis for Molecular-Orbital Studies of Organic Molecules. J. Chem. Phys. 1971, 54, 724 10.1063/1.1674902.

[ref43] Behn A.; Zimmerman P. M.; Bell A. T.; Head-Gordon M. Efficient Exploration of Reaction Paths via a Freezing String Method. J. Chem. Phys. 2011, 135 (22), 224108 10.1063/1.3664901. 22168681

[ref44] Fukui K. Formulation of the Reaction Coordinate. J. Phys. Chem. 1970, 74 (23), 4161–4163. 10.1021/j100717a029.

