# Supplementary material for: Accelerated Multiphosphorylated Peptide Synthesis
Source: Org Process Res Dev. 2022 Jul 12;26(8):2492–7. doi: 10.1021/acs.oprd.2c00164 (PMC9397535; doi:10.1021/acs.oprd.2c00164)
Supplement: Supplementary file 1 — op2c00164_si_001.pdf [file op2c00164_si_001.pdf]

Supporting information

Accelerated multi phosphorylated peptide synthesis

Dana Grunhaus[a], Estefanía Rossich Molina,[a,b] Roni Cohen[a], Tamar Stein,[a,b] Assaf Friedler[a] and Mattan Hurevich[a]

[a] The Institute of Chemistry, The Hebrew University of Jerusalem, Edmond J. Safra Campus, Givat Ram, Jerusalem, 91904, Israel

[b] The Fritz Haber Research Center for Molecular Dynamics, The Hebrew University of Jerusalem.

*corresponding authors. Emails: mattan.hurevich@mail.huji.ac.il ,assaf.friedler@mail.huji.ac.il

Contents

1. Materials and methods 2

 1.1 Materials 2

 1.2 Instruments 2

2. Preparation of Reagents 3

 2.1 Reagents for coupling 3

 2.2 Bases for Fmoc deprotection 3

3. HPLC analysis of base screening chromatograms 3

4. Syntheses conditions and cycles 4

 4.1 Conditions 4

 4.2 HPLC analyses for B2R-5p synthesis via MW-SPPS and AMPS 5

 4.3. Chromatograms and ESI-MS data of MPP library 6

5. Computational details: Modeling Fmoc deprotection and β-elimination processes using piperidine base 13

1. Materials and methods

1.1 Materials

All Fmoc-amino acids were obtained from GL Biochem. (Shanghai), or Matrix Innovation (Quebec City, Canada), with the following side chain protecting groups: Arg(Pbf), Asp(OtBu), Asn(Trt), Glu(OtBu), Gln (Trt), His(Trt),Ser(tBu), Thr(tBu), Cys(Trt), Lys(Boc), Tyr(tBu). (Pbf = 2,2,4,6,7- pentamethyl-2,3-dihydrobenzofuran-5-sulfonyl). Rink Amide resin (0.48 mmol/g) was purchased from Matrix Innovation. 1-[Bis(dimethylamino)methylene]-1H-1,2,3-triazolo[4,5-b]pyridinium 3-oxide hexafluorophosphate (HATU), was purchased from Luxembourg Biotechnologies Ltd. (Rehovot, Israel). 1,8-Diazabicyclo(5.4.0)undec-7-ene (DBU) was purchased from Merck (Rehovot, Israel). Triisopropylsilane (TIPS), 98% was purchased from Alfa Aesar. Piperazine and Morpholine, 99% extra pure, were purchased from Acros Organics (Holland Moran Israel). Solvents: N,N-dimethylformamide (DMF), dichloromethane (DCM), acetonitrile (ACN), N,N-diisopropylethyl amine (DIEA), Trifluoroacetic acid (TFA), piperidine (Pip), methanol (MeOH), and diethyl ether were purchased from BioLab. (Jerusalem, Israel) and were peptide synthesis or HPLC-grade.

1.2 Instruments

Semi-preparative RP-HPLC were performed on a Waters 150Q LC system using a C18 column (Phenomenex Luna 5 µm, 100 Å 21.2 × 250 mm) at a flow rate of 15 mL/min and recorded at 220 nm Analytical RP-HPLC were performed on Merck Hitachi HPLC with a reverse-phase C18 analytical column. (Merck Purospher STAR RP-18 endcapped LiChroCART® 250-4.6; 5 µm, or Zorbax RX-C18 150-4.6; 5 µm) with flow rate of 1 mL/min and recorded at 220 nm Linear gradients of ACN (with 0.1 % TFA, buffer B) in water (with 0.1 % TFA, Buffer A) were used for all systems to elute bound peptides.

ESI-MS was performed on LCQ Fleet Ion Trap mass spectrometer (Thermo Scientific). Peptide masses were calculated from the experimental mass to charge (m/z) ratios from the observed multiply-charged species of a peptide.

Deconvolution of the experimental MS data was performed with the help of MagTran v1.03 software.

2. Preparation of Reagents

2.1 Reagents for coupling

Amino acid solutions were prepared by adding a solution of 3 equiv. Fmoc-protected amino acids in 1 mL DMF. An activator solution was prepared by dissolving 2.9 equiv. of HATU in 1 mL of DMF per coupling. A base solution was prepared by dissolving 8 equiv. of DIEA in 1 mL DMF per coupling.

2.2 Bases for Fmoc deprotection

Solutions of 0.5 and 5% and 20% (V/V) piperidine in DMF were prepared.
A solution of 1% of piperazine (W/V) in DMF,
A solution of 10% of Morpholine (V/V) in DMF

3. HPLC analysis of base screening chromatograms

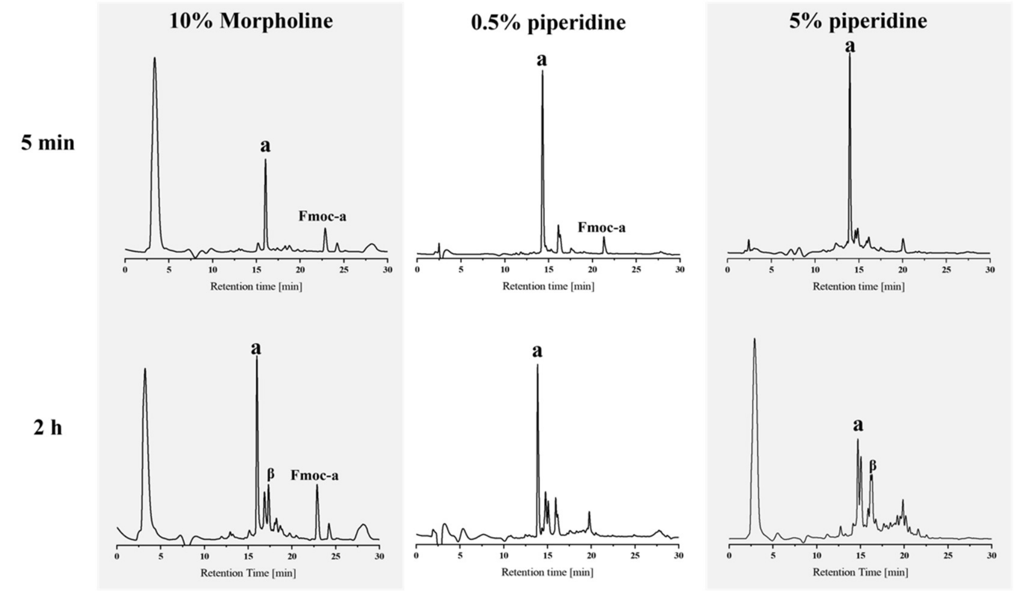

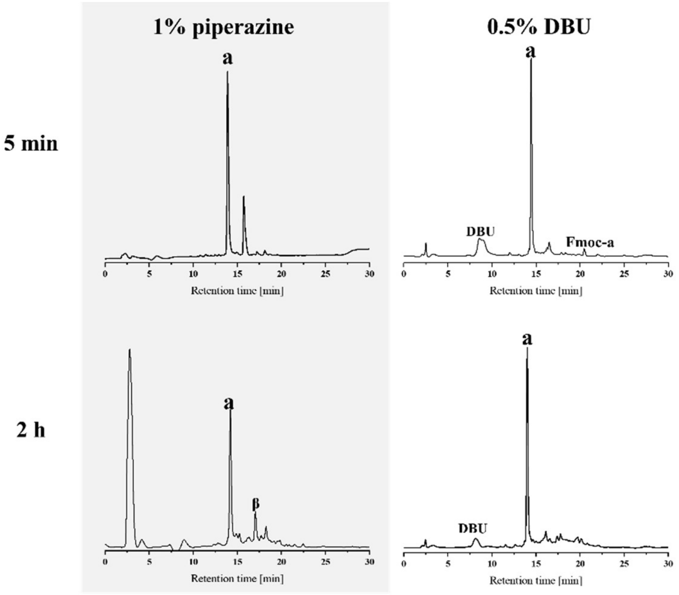

Figure S1. Analytical HPLC chromatograms of the Fmoc deprotection from peptide **Fmoc-a** using different bases and concentrations. **a**= pSLGLGLG peptide. **Fmoc-a** = Fmoc-pSLGLGLG, **β**= dephosphorylated β-elimination product or its 3-(1-piperidinyl alanine) adduct. *residual DBU eluted with the crude peptide as reported previously.[1]

4. Syntheses conditions and cycles

4.1 Conditions

Table 1S: MW-SPPS of **B2R-5p** via liberty blue synthesizer (CEM)

**Reaction**	**Conditions**
Fmoc deprotection	6 mL of 20% piperidine/DMF 10 min
Wash	5 X 6 mL DMF
Coupling	The resin was incubated with 5 mL of DMF solution containing: 5 equiv. AA, 5 equiv. HATU, and 10 equiv. DIEA for 5 min.<br>Mixing: N_2 bubbling 2 s on 3 s off
Wash	5 X 6 mL DMF

Table 2S: AMPS of **B2R-5p**

**Reaction**	**Conditions**
Fmoc deprotection	3 mL of 0.5% DBU/DMF 10 s
Wash	2 X 3 mL DMF. Total 15 s
Coupling	The resin was incubated with 3 mL of DMF solution containing: 3 equiv. AA, 2.9 equiv. HATU, and 8 equiv. DIEA for 1 min.<br>Mixing: 1200 rpm
Wash	2 X 3 mL DMF. Total 15 s

4.2 HPLC analyses for B2R-5p synthesis via MW-SPPS and AMPS

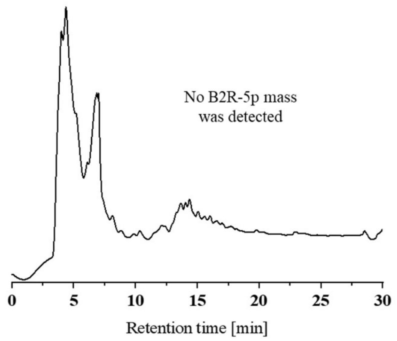

Figure S2. Chromatogram of crude **B2R-5p** synthesized via MW-SPPS. A peak associated with **B2R-5p** was not detected.

B2R-5p (366-375) - pS M G pT L R pT pS I pS

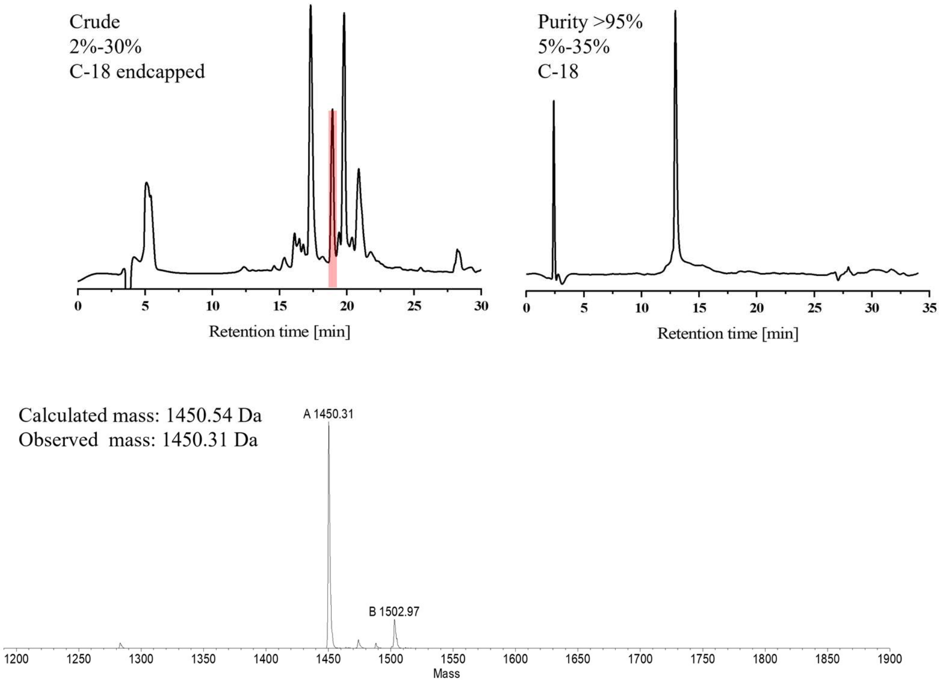

Figure S3. Chromatogram of crude (left) and pure (right) **B2R-5p** synthesized via AMPS. ESI-MS (bottom) of the isolated peptide.

4.3. Chromatograms and ESI-MS data of MPP library

V2R-5p (362-371) - pS pS pS L A K D pT pS S

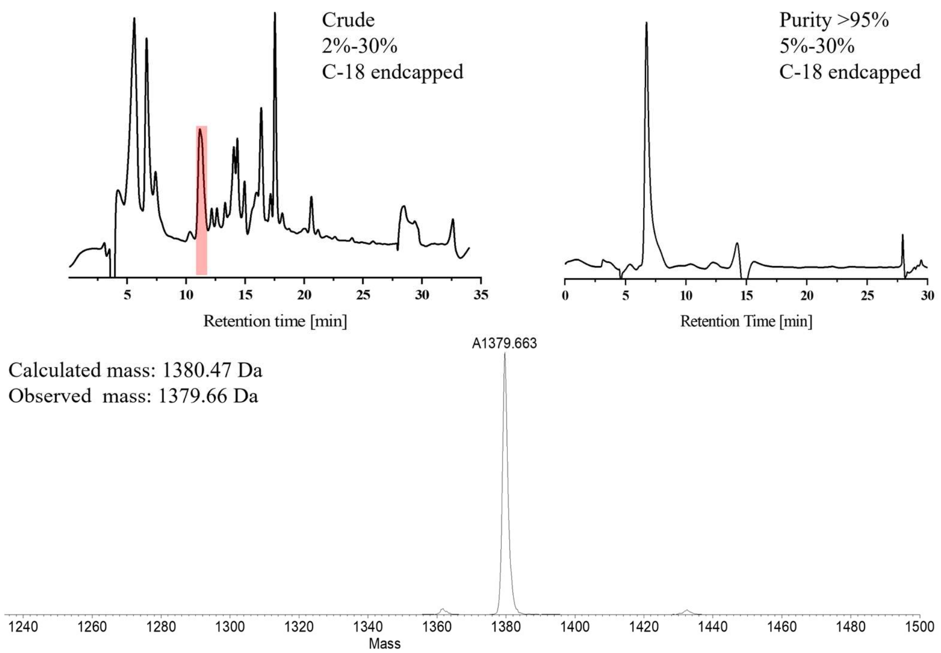

Figure S4. Chromatogram of crude (left) and pure (right) **V2R-5p** synthesized via AMPS. ESI-MS (bottom) of the isolated peptide.

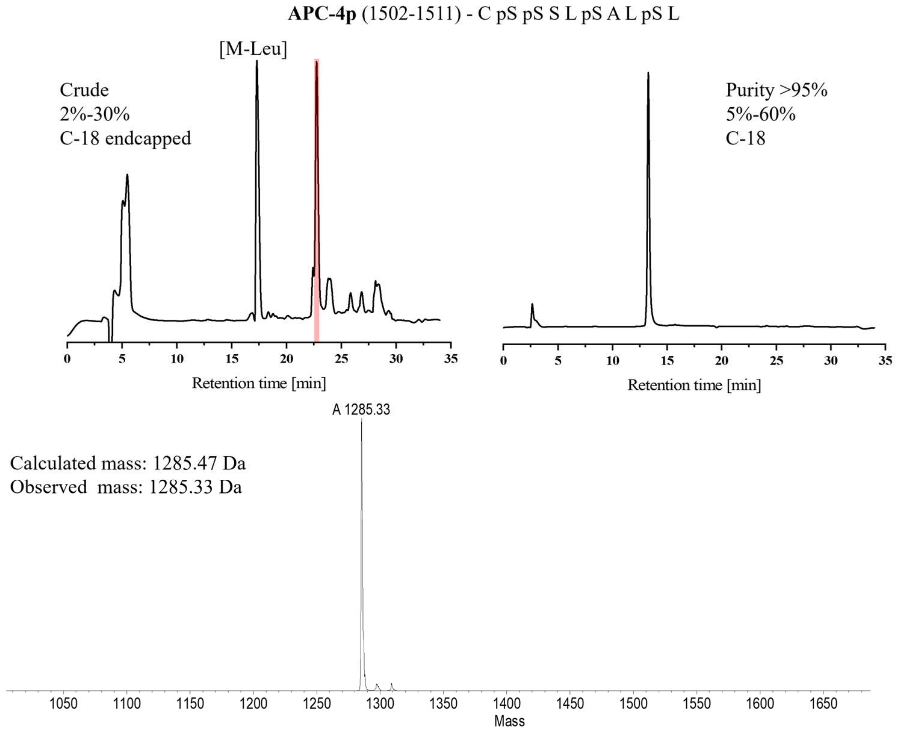

Figure S5. Chromatogram of crude (left) and pure (right) **APC-4p** synthesized via AMPS. ESI-MS (bottom) of the isolated peptide.

P53-5p (6-20) - pS D P pS V E P P L pS Q E pT F pS

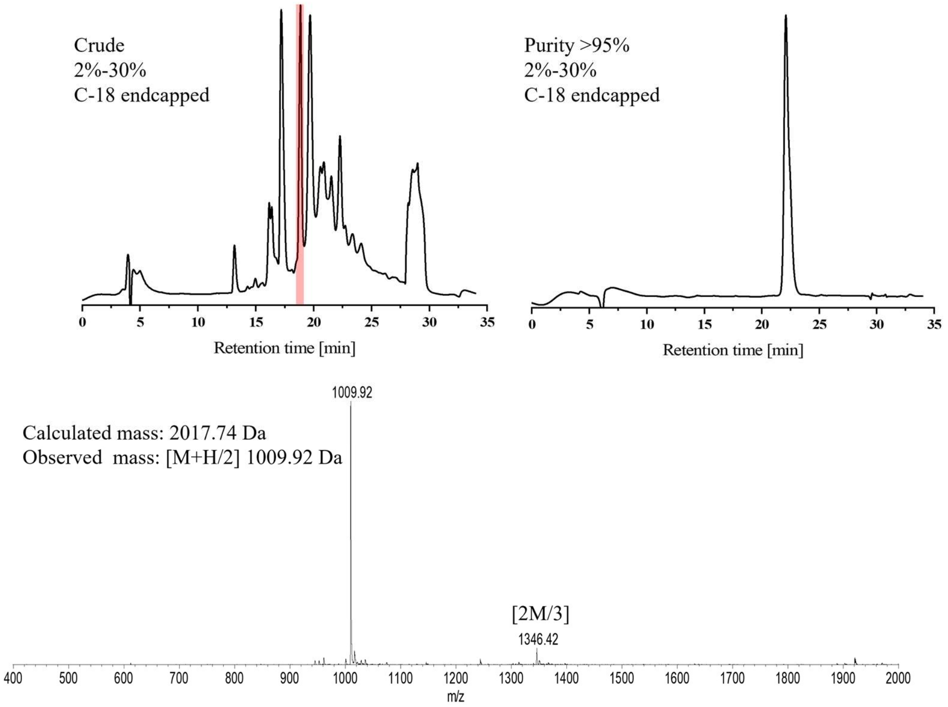

Figure S6. Chromatogram of crude (left) and pure (right) **P53-5p** synthesized via AMPS. ESI-MS (bottom) of the isolated peptide.

Vim-4p (22-30) - L L L pS R P pS pS pS R S

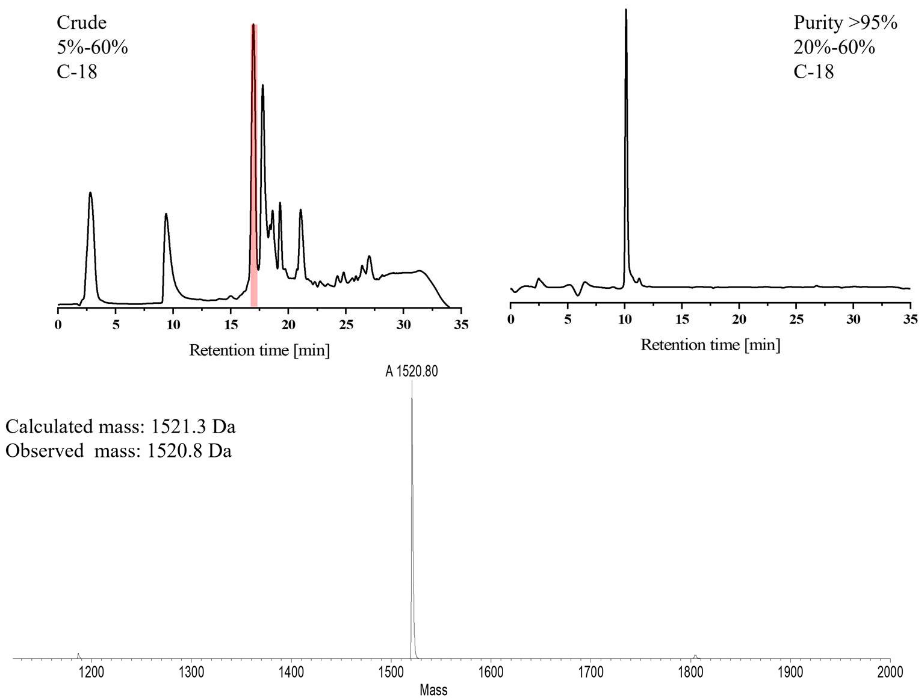

Figure S7. Chromatogram of crude (left) and pure (right) **Vim-4p** synthesized via AMPS. ESI-MS (bottom) of the isolated peptide.

Tau-6p (515-527) - pS pS P G pS P G pT P G pS R pS L L L

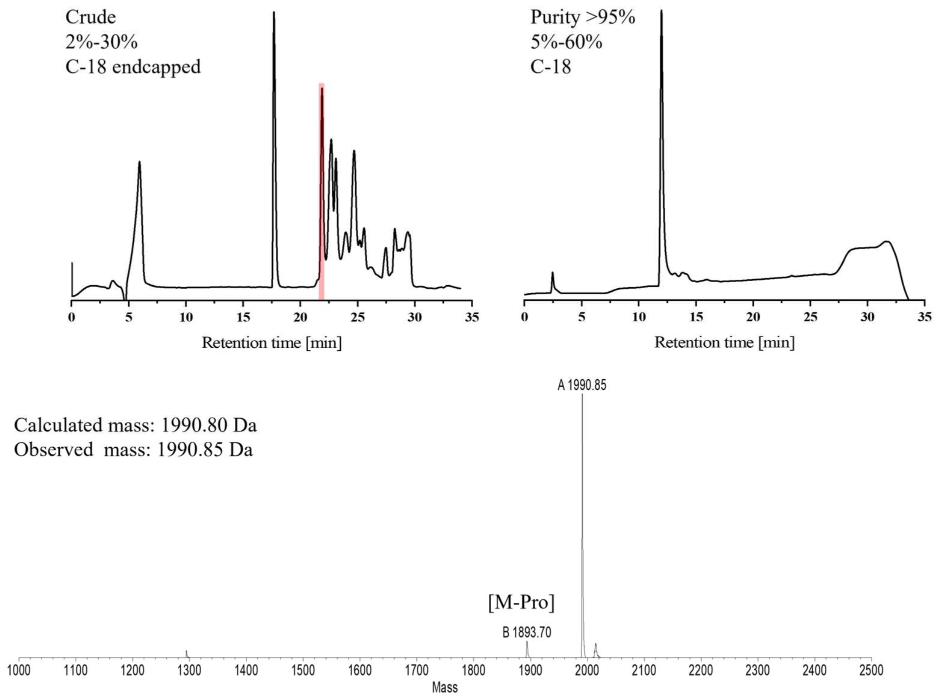

Figure S8. Chromatogram of crude (left) and pure (right) **Tau-6p** synthesized via AMPS. ESI-MS (bottom) of the isolated peptide.

pLam-4p (404-411) - pS H pS pS Q pT Q G L L L

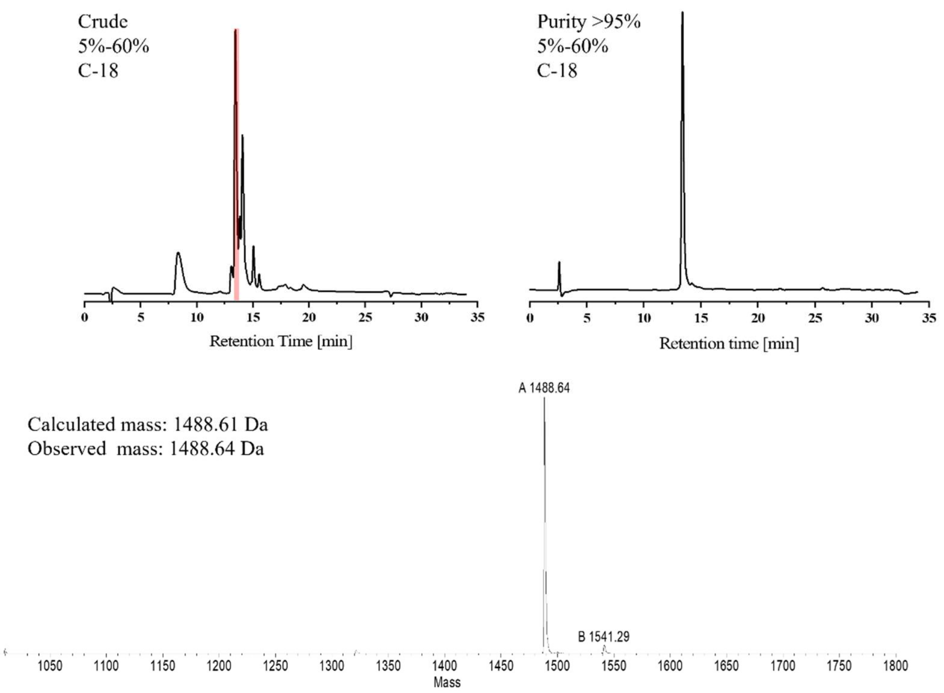

Figure S9. Chromatogram of crude (left) and pure (right) **pLam-4p** synthesized via AMPS. ESI-MS (bottom) of the isolated peptide.

FFAR4-5p (346-360) - L pT D pT pS V K R N D L pS I I pS

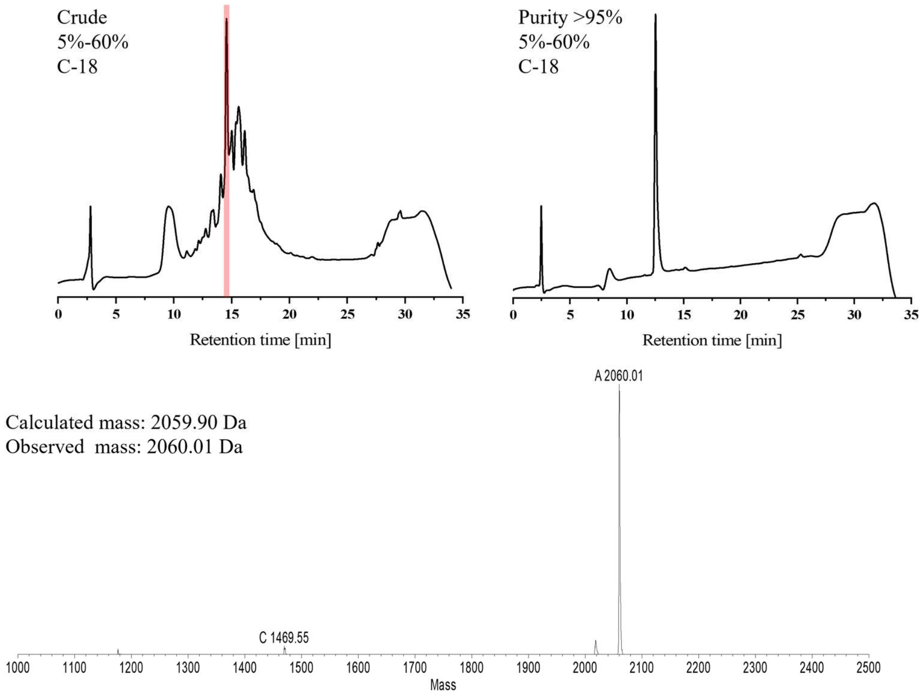

Figure S10. Chromatogram of crude (left) and pure (right) **FFAR4-5p** synthesized via AMPS. ESI-MS (bottom) of the isolated peptide.

5. Computational details: Modeling Fmoc deprotection and β-elimination processes using piperidine base

Fmoc deprotection and β-elimination reactions were also modeled using piperidine as the base. As done with DBU, the Fmoc deprotection reaction was studied from a model system consisting of one Fmoc-Ser/(HPO_3Bzl)-OH and one piperidine molecule. We found similar mechanisms with piperidine to those observed with DBU. The Fmoc deprotection is a two-step process, shown in Figure S11(i) and S12 (i). The first step is a proton transfer from the Fmoc group in Fmoc-Ser/(HPO_3Bzl)-OH to the piperidine (structure i-a), which results in a bond-breaking leading to [CO_2-Ser/(HPO_3Bzl)-OH]$^-$ and piperidineH$^+$ (i-c). The barrier for this step is calculated to be 13.8 kcal/mol. In the second step, a piperidineH$^+$ molecule transfers a proton to the N of the Ser, resulting in CO_2 departure and formation of pSer (i-f). The energy barrier for this step is 0.7 kcal/mol. To model the β-elimination reaction with piperidine, we used Ser/(HPO_3Bzl)-OH with one piperidine as a model system; the first step (not shown) is a barrier-less proton transfer from the (HPO_3Bzl)-OH group to the piperidine base, which results in [Ser/(PO_3Bzl)-OH]$^-$ and piperidineH$^+$. As in the DBU case, an additional piperidine molecule is needed to model the next reaction step. Thus, our starting point is [Ser/(PO_3Bzl)-OH]$^-$, piperidine and piperidineH$^+$ to maintain the neutrality of the system, as shown in Figure S11(ii-a). The second step consists of a proton being

transferred from the Cα of the [Ser/(PO_3Bzl)-OH]$^-$ to the piperidine resulting in the dephosphorylation of the Ser (ii-c). The energy barrier for this step is 16.6 kcal/mol.

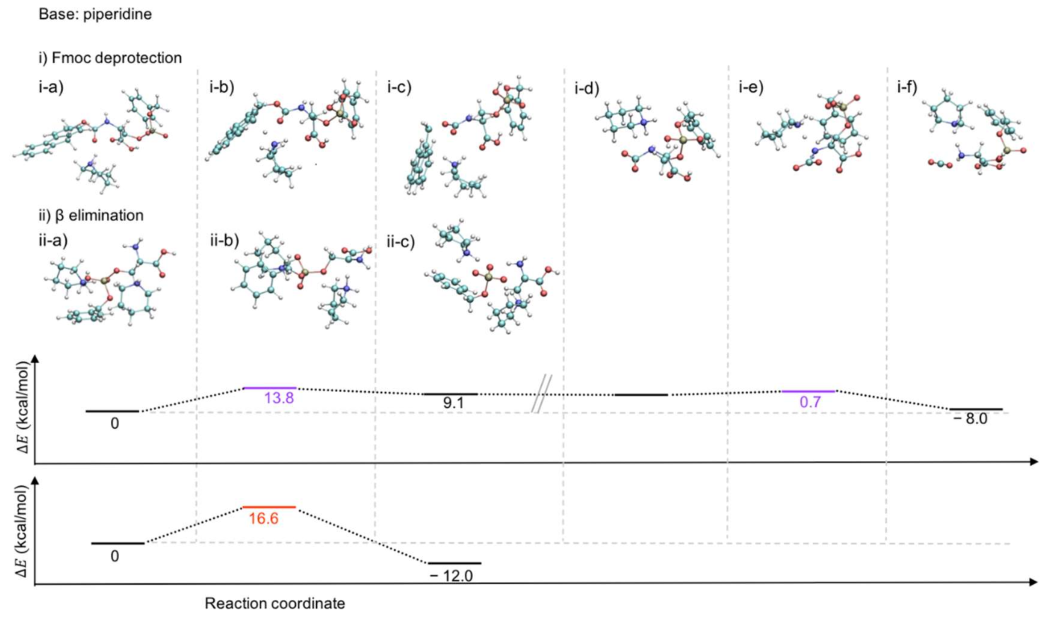

Figure S11. PES representing Fmoc deprotection **(i)** and β-elimination **(ii)** processes with piperidine. Above each bar, the associated chemical structure is presented. Black bars represent minima on the PES, and purple/red bars represent transition states. ΔE values are calculated with respect to the initial structures (i-a/ii-a). In the case of i-d), the ΔE value is omitted as, when going from i-c) to i-d), we have removed from the model system Fmoc-Ser/(HPO_3Bzl)-OH the molecular fragment that has dissociated. The ΔE for i-e) and i-f) are relative to the energy of i-d).

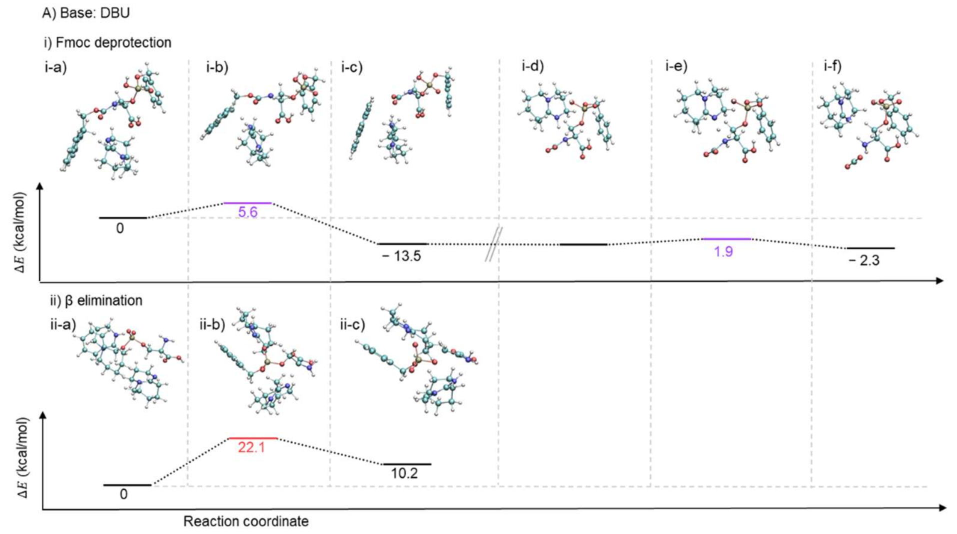

Figure S12. PES representing Fmoc deprotection **(i)** and β-elimination **(ii)** processes with DBU. Above each bar, the associated chemical structure is presented. Black bars represent minima on the PES, and purple/red bars represent transition states. ΔE values are calculated with respect to the initial structures (i-a/ii-a). In the case of i-d), the ΔE value is omitted as, when going from i-c) to i-d), we have removed from the model system Fmoc-Ser/(HPO_3Bzl)-OH the molecular fragment that has dissociated. The ΔE for i-e) and i-f) are relative to the energy of i-d).

References

(1) Attard, T. J.; O’Brien-Simpson, N. M.; Reynolds, E. C. Identification and Suppression of β-Elimination Byproducts Arising from the Use of Fmoc-Ser(PO3Bzl,H)-OH in Peptide Synthesis. *Int J Pept Res Ther* **2009**, *15*, 69–79. https://doi.org/10.1007/s10989-008-9165-9.
